# Hydropersulfides promote angiogenesis and preserve vascular function

**DOI:** 10.1016/j.redox.2026.104192

**Published:** 2026-04-30

**Authors:** Reece J. Lamb, Fifi S. Ibrahim, Vinayak S. Khodade, Scott P. Davies, Kayleigh Griffiths, Tsuyoshi Takata, Jinjing Gu, Tetsuro Matsunaga, Roger J. Grand, Francesca M. Nichols, Alice J. Barton, Aisah A. Aubdool, Masanobu Morita, Hozumi Motohashi, Ming Xian, Takaaki Akaike, John P. Toscano, Melanie Madhani

**Affiliations:** a Department of Cardiovascular Sciences, School of Medical Sciences, College of Medicine and Health, University of Birmingham, United Kingdom; b Department of Chemistry, Johns Hopkins University, Baltimore, MD, USA; c Deparment of Inflammation and Immunotherapy, School of Infection, Inflammation, and Immunology, College of Medicine and Health, University of Birmingham, United Kingdom; d Department of Redox Molecular Medicine, Tohoku University Graduate School of Medicine, Sendai, 980-8575, Japan; e William Harvey Research Institute, Faculty of Medicine and Dentistry, Barts & The London Hospitals, Queen Mary University of London, United Kingdom; f Department of Medical Biochemistry, Tohoku University Graduate School of Medicine, Sendai, 980-8575, Japan; g Department of Chemistry, Brown University, Providence, RI, USA; h National Heart Research Institute Singapore, National Heart Centre Singapore, Singapore

## Abstract

Hydropersulfides (RSSH) are increasingly recognized as key mediators in redox signalling in mammalian cells, although their physiological functions, especially in angiogenesis, remain unknown. Direct mechanistic investigation of RSSH has been challenging due to their instability and high reactivity. To address this, donors that release RSSH in a controlled manner have been developed, enabling investigation of its biological roles. Herein, we employed thiol- and enzyme-activated RSSH donors along with mutant mouse models deficient in the Cys–SSH–producing enzyme cysteinyl-tRNA synthetase/cysteine persulfide synthase (CARS2/CPERS), to examine their role in vascular angiogenesis. We demonstrate that RSSH promotes angiogenesis via the CARS2/CPERS signalling axis and activation of the Akt-eNOS and NO-cGMP pathway. We also demonstrate that nitric oxide (NO) signalling in resistance vessels requires an intact CARS2/CPER2 pathway. These findings suggest that RSSH plays an important role in regulating angiogenesis and vascular tone.

## Graphical abstract

**Figure:**
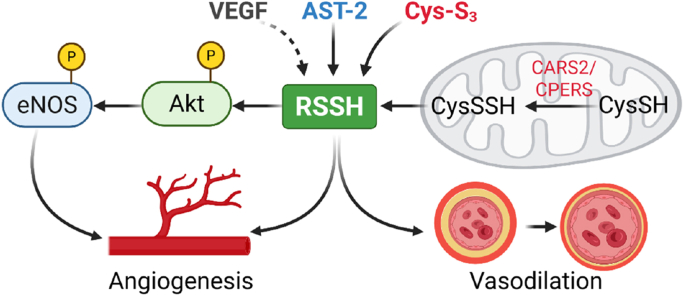

## 1 Introduction

Angiogenesis, the formation of new blood vessels from existing vasculature, is crucial for embryonic development, wound healing and restoring blood flow after injury [1]. However, dysregulated angiogenesis contributes to the pathogenesis of numerous disorders. Excessive angiogenesis can drive tumour growth and chronic inflammatory diseases such as rheumatoid arthritis, whereas insufficient angiogenic responses impair tissue regeneration in conditions such as peripheral vascular and ischaemic heart disease [2].

Angiogenesis is a complex, multi-step process that involves endothelial cell proliferation, migration and differentiation [3]. These endothelial activities drive vessel sprouting, branching, and intussusceptive growth. The orchestration of angiogenesis is tightly regulated by the fine balance between pro- and anti-angiogenic factors, with vascular endothelial growth factor (VEGF) as a central mediator. VEGF stimulation leads to the production of the gasotransmitters, hydrogen sulfide (H_2_S) and nitric oxide (NO), both of which are key mediators of angiogenic signalling [4,5]. H_2_S potentiates the NO-cGMP dependent pathway by activating the Akt mediated phosphorylation of endothelial nitric oxide synthase (eNOS), which promotes endothelial cell growth and migration [6]. While NO signalling is well-characterised, the mechanisms by which H_2_S regulate angiogenesis remain unclear, suggesting the involvement of additional, yet unidentified mediators.

Recent findings suggest that some biological effects attributed to H_2_S may, in fact, arise partly from its conversion to other reactive sulfur species (RSS), particularly small molecule hydropersulfides (RSSH) and polysulfides (RSS*_n_*SR, *n* > 1). Among these, cysteine hydropersulfide (Cys-SSH), glutathione hydropersulfide (GSSH), and coenzyme A-persulfide (CoA-SSH) are prevalent in mammalian cells [7,8]. These RSSH can form from H_2_S reaction with thiol-containing molecules like cysteine (Cys-SH), glutathione (GSH), and CoA-SH in the presence of oxidants or independently through reactions with other sulfur sources [8,9]. RSSH species exhibit strong antioxidant capacity and confer cytoprotective effects under physiological and stress-related conditions [[10], [11], [12]].

Previous studies have also identified mitochondrial cysteinyl-tRNA synthetase (CARS2), as the primary enzymatic source of endogenous Cys-SSH [13]. Furthermore, we have shown that Cys-SSH and related polysulfide species are endogenously generated in the vasculature [14]; however, the physiological relevance of RSSH, particularly in the regulation of cardiovascular function, is not fully understood. Specifically, the role of RSSH, in particular CARS2/cysteine persulfide synthase (CPERS)-derived Cys-SSH, in angiogenesis remains unknown.

Despite growing recognition of RSSH as pivotal mediators of redox signalling, direct mechanistic interrogation of these species remains challenging due to their inherent instability and high reactivity. The dual nucleophilic and electrophilic nature of RSSH renders them prone to rapid disproportionation, producing polysulfides, and H_2_S [15]. To overcome these limitations, several small-molecule donors have been developed that release RSSH in a controlled manner [[16], [17], [18]], providing valuable tools for probing their physiological functions. A few studies have begun to explore the role of RSSH in angiogenesis. For example, polymeric per/polysulfide delivery systems, such as BDP-NAC fiber mats and manganese porphyrin (MnPMC) -containing micelles, promote tube formation in endothelial cells [19,20]. However, mechanistic insight into RSSH-mediated angiogenesis remains limited. Here, we employed two structurally and mechanistically distinct RSSH-releasing donors, in combination with thiol control analog and genetically modified mouse models deficient in endogenous Cys-SSH biosynthesis, to investigate the role of RSSH in the regulation of angiogenesis. This integrative approach suggests that endogenous RSSH signalling plays an important role in maintaining angiogenesis, highlighting a previously unrecognized dimension of sulfur redox biology in vascular homeostasis.

## 2 Materials and methods

### 2.1 Ethics

All murine studies complied with the UK Animals (Scientific Procedures) Act of 1986, EU Directive 2010/63/EU, and were approved by the University of Birmingham Animal Welfare and Ethical Review Body. The experimental approaches for the different *ex vivo* experiments are described in detail below.

### 2.2 Chemicals and reagents

Cysteine trisulfide (Cys-S_3_; also known as thiocysteine) and alkylsulfenyl thiocarbonate (AST-2) were synthesized as previously described [21,22] and freshly prepared each day in DMSO:water (<0.001%) prior to use. All other chemicals were of the highest purity commercially available and obtained either from Merck, Fisher Scientific or VWR, unless specified elsewhere.

### 2.3 Cell culture

Human umbilical vein endothelial cells (HUVECs; PromoCell C-12200) were cultured in endothelial cell growth media (PromoCell, C22010) supplemented with 1% penicillin/streptomycin (Gibco). Cells were maintained at 37 °C in a humidified incubator (95% O_2_/5% CO_2_), as previously described [23]. Passages 1-5 were used for all experiments.

### 2.4 Cell proliferation and viability assay

For the determination of proliferation and viability, HUVECs were seeded at 5 x 10^3^ cells per well in 96-well plates and cultured overnight in endothelial cell growth medium at 37 °C, (95% O_2_/5% CO_2_). The next day, cells were serum-starved (0.5% FBS) overnight under the same conditions. Following serum-starvation, HUVECs were treated with vehicle control, VEGF (50 ng/ml; Bio-Techne; NBP3-07101), Cys-S_3_, AST-2, or thiol controls for 24, 48 or 72 h in endothelial cell growth medium. Cell proliferation was measured using BrdU assay (Roche Diagnostics). Viability under these conditions was confirmed using the trypan blue exclusion method, as previously described [24]. All treatments were performed in triplicate.

### 2.5 Wound scratch assay

To evaluate the effects of exogenous RSSH donors on HUVEC migration, a scratch wound assay was performed as previously described [25]. HUVECs were seeded at 2.0 x 10^4^ cells/well in 96-well plates and cultured to confluence. After 24 h at 37 °C in a humidified incubator (95% O_2_/5% CO_2_), HUVECs were serum-starved (0.5% FBS) for 4 h. The HUVEC monolayer was then scratched (∼0.5 mm width) using a sterilised endothelial wound maker tool (Incucyte® 96-well Woundmaker Tool, Sartorius). Detached cells were removed by washing twice with culture medium, which was then replaced with fresh endothelial growth medium. HUVECs were treated with VEGF (50 ng/ml) vehicle control (0.0002 - 0.2% DMSO), or varying concentrations of Cys-S_3_, AST-2 or thiol controls for 24 h. In separate experiments, HUVECs were pre-treated with l-NAME (NOS inhibitor, Nω-Nitro-l-arginine methyl ester hydrochloride; 100 μM or 500 μM) or LY294002 (PI3K inhibitor; 1 μM or 10 μM) for 30 min, followed by incubation with VEGF (50 ng/ml), Cys-S_3_ (10 μM or 100 μM), or AST-2, (10 μM or 100 μM) for 24 h. Plates were immediately placed into an Incucyte live-cell imaging system (Sartorius, Bioscience) at 10× magnification, and images of wound closure were captured every hour over a 24 h period.

### 2.6 Detection of polysulfide (sulfane sulfur) species formation in HUVECs

RSSH formation was assessed employing two complementary methods: First, qualitative monitoring using sulfane sulfur specific fluorescent probe SSP4 [26], and second, quantitation of polysulfide (sulfane sulfur), including both inorganic and organic polysulfides by triphenylphosphine based-liquid chromatography-mass spectrometry LC-MS/MS [27]. HUVECs were seeded into 96-well plates at 1.5 x10^4^ cells/well and cultured until confluency. After 24 h, cells were serum-starved for 4 h at 37 °C in a humidified incubator (95% O_2_/5% CO_2_). Following starvation, the medium was replaced with endothelial cell growth medium, and cells were incubated with VEGF (50 ng/ml), Cys-S_3_, AST-2 or thiol control at concentrations of 10 μM or 100 μM for 24 h. The next day, cells were either assessed with SSP4 or LC-MS/MS analysis, respectively.

#### 2.6.1 SSP4 analysis

Cells were fixed with 4% paraformaldehyde for 10 min at room temperature. After fixation, HUVECs were washed with PBS, incubated with 150 μM CTAB (cetrimonium bromide) and 20 μM SSP4 for 30 min, and washed again with PBS. Fresh PBS was added prior to imaging. Fluorescence imaging was performed using a Zeiss Cell Discoverer 7 system. Fluorescence intensity was quantified using Cell Profiler v 4.2.8 (Supplementary Figure 1) [28]. Measurements taken for experimental conditions were corrected for the value obtained for the negative control.

#### 2.6.2 Triphenylphosphine based-polysulfide measurement

The protocol is based on a previously developed method [27], with some modifications. Briefly, the chloroform extract of HUVECs was reacted with tris(2,4,6-trimethoxyphenyl)phosphine - (mPh)_3_P (Sigma, 392081) in toluene. Subsequently, the mixture was analysed using LC-MS/MS (Shimadzu 8060). The LC settings were as follows: Eluent A consisted of 0.1% formic acid (FA), and eluent B consisted of 0.1% FA in methanol. The column used was a YMC-Triart C18 column with a 5 × 2.0 mm internal diameter and a 3 μm particle size. The column temperature was maintained at 40 °C. The flow rate was set at 0.2 mL/min. The gradient program was as follows: from 0 to 2 min, eluent B was kept at 1%; from 2 to 7 min, eluent B was increased to 80%; from 7 to 12 min, eluent B was increased to 99%; from 12 to 15 min, eluent B was kept at 99%; and from 15 to 22 min, the system was re-equilibrated to the initial conditions.

### 2.7 Western blotting

HUVEC were cultured to confluence in 6-well plates and serum-starved overnight. HUVECs were then treated with vehicle control, Cys-S_3_, AST-2, or thiol controls (10 and 100 μM) for 5 min, 30 min, 1 h, 3 h, 8 h or 24 h. After treatment, cells were washed twice with PBS and gently aspirated. RIPA buffer (50 μl/well, Abcam) supplemented with protease and phosphatase inhibitors was added (Roche). Cells were scraped, lysates pooled and centrifuged at 13,000 rpm for 10 min at 4 °C. Supernatants were collected, and protein concentration was determined by using a Bradford assay (Bio-Rad Laboratories). Equal amounts of protein (5 μg/well for AKT or 25 μg/well for eNOS) were mixed with 5% Laemmli buffer (Bio-Rad Laboratories) containing β-mercaptoethanol and boiled at 95 °C for 5 min. Samples were separated by 8-10% SDS-PAGE and transferred to PVDF membranes (Amersham Hybond® Western blotting membrane; 1060029 Cytiva). Membranes were blocked (3% milk or 5% BSA powder in PBS) and incubated overnight with primary antibodies: *p*-Akt^ser473^ (1:5000; 4060S; Cell Signalling), total AktT (1:5000; 9272; Cell Signalling), *p*-eNOS^ser1177^ (1:1000; 9574S Cell Signalling), total eNOS (1:5000; 610297 BD Biosciences), vasodilator-stimulated phosphoprotein (VASP) at ser-239 (*p*-VASP^ser239^; 1:1000; 3114 Cell Signalling), total VASP (1:10,000; 3132 Cell Signalling) and GAPDH (1:1000; 97166S Cell Signalling) as loading control. After washing, the membranes were incubated with HRP-conjugated secondary anti-mouse (554002 Fisher Scientific) or anti-rabbit antibodies (1:10,000; 7074S Cell Signalling) for all, except for *p*-eNOS^ser1177^ – 1:1000 and total VASP – 1:5000) and developed using enhanced chemiluminescence (ECL) reagent (32106 Thermo Fisher Scientific). Densitometric analysis of digitised immunoblots was performed using Adobe Photoshop 2021.

### 2.8 cGMP determination

HUVECs were seeded at 2 x 10^4^ cells per well in 24-well plates and cultured overnight in endothelial cell growth medium at 37 °C (95% O_2_/5% CO_2_). The following day, cells were serum-starved overnight in medium containing 0.5% FBS under the same conditions. After serum-starvation, HUVECs were re-suspended in endothelial cell growth medium containing 1 mM 3-Isobutyl-1-methylxanthine for 30 min (IBMX; Merck Life Science) followed by treatment with vehicle control, VEGF (50 ng/ml), 100 μM Cys-S_3_, 100 μM AST-2, or positive control NO donor, spermine-NONOate (100 μM SPER-NO; Cayman Chemical) for 1 h. Intracellular cGMP levels were then measured using a commercially available ELISA kit (ab323639).

### 2.9 In vitro tube formation assay

A 24-well plate was coated with 200 μl of growth factor-reduced Geltrex (A1413202 Gibco) according to manufacturer's guidelines. Plates were incubated for 30 min at 37 °C to allow gel polymerisation. HUVECs (6 x 10^4^ per/well) were seeded onto the Geltrex in endothelial cell growth medium. Tubule formation was assessed after treatment with VEGF (50 ng/ml; positive control), Cys-S_3_, AST-2, or thiol control (negative control) at varying concentrations (0.1-100 μM) over a 24h period. Tubule formation was quantified by measuring the tube length, using 4× magnification inverted phase-contrast microscope (Olympus IX70).

### 2.10 Aortic sprouting assay

Male C57BL/6, *Cars2*^*+/−*^, *Cars2*^*Aink/+*^ and their WT littermates (25-30g) were anticoagulated with 0.1 IU sodium heparin and anesthetized using sodium pentobarbital (300 mg/kg i.p). Thoracic aortas were excised, cleared of fat, and flushed with Opti-MEM (Gibco) through the lumen to remove residual blood. Aortic rings of ∼0.5 mm were cut and embedded in a 1 mg/ml type 1 rat collagen matrix (A10483-01 Gibco) [29]. After a 1 h incubation at 37 °C (95% O_2_/5% CO_2_), 150 μl of Opti-MEM containing 2.5% FBS, 1% penicillin/streptomycin, with or without 50 ng/ml VEGF, Cys-S_3_, AST-2, or thiol control (10 or 100 μM) were added to each well. In separate experiments, aortic rings were pre-treated with l-NAME (500 μM) or LY294002 (10 μM) for 30 min, followed by incubation with 50 ng/ml VEGF, 100 μM Cys-S_3_, or 100 μM AST-2 for 7 days. Culture medium and treatments were refreshed every 3 days. On day 7, rings were fixed and endothelial cells were stained with Bandeiraea Simplicifolia Isolectin B4 (BSI–B_4_; L2895 Merck) FITC conjugate. Immunofluorescence Z-stack images of microvessel sprouts were acquired using Cell Discoverer 7 (Zeiss) and sprout numbers were quantified by two independent, blinded analysts using image J.

### 2.11 Functional studies on isolated blood vessels for myograph analysis

Thoracic aorta (conduit vessels) and second-order mesenteric resistance vessels were isolated from *Cars2*^*+/−*^, *Cars2*^*Aink/+*^ and their wild-type (WT) littermates (25-30g). Vessels were cleaned of adipose and connective tissue and then mounted in a tension myograph (Danish Myo Technology, Hinnerup, Denmark) filled with Krebs-Henseleit buffer (KHB; 37 °C, pH 7.4) and gassed with 95% O_2_/5% CO_2_, as previously described [14,30]. After equilibration, vascular reactivity was normalized. Submaximal contraction was induced using phenylephrine for aortic rings or U46619 (Bio-Techne) for mesenteric arteries, followed by cumulative concentration-response curve (1 nM-10 μM) to SPER-NO. Results were expressed as a percentage of the stable pre-contractile tone.

### 2.12 Statistical analysis

All data are presented as means ± standard error of the mean (SEM), where *n* is the number of animals or experiments. Statistical analysis was conducted by GraphPad Prism software (version 10.4.1) using one-way ANOVA and two-way ANOVA for comparisons, followed by Dunnett's, Tukey's or Šídák's multiple comparisons test. P ≤ 0.05 was considered significant.

## 3 Results

### 3.1 Development of hydropersulfide donors and thiol tools

**Fig. 1:**
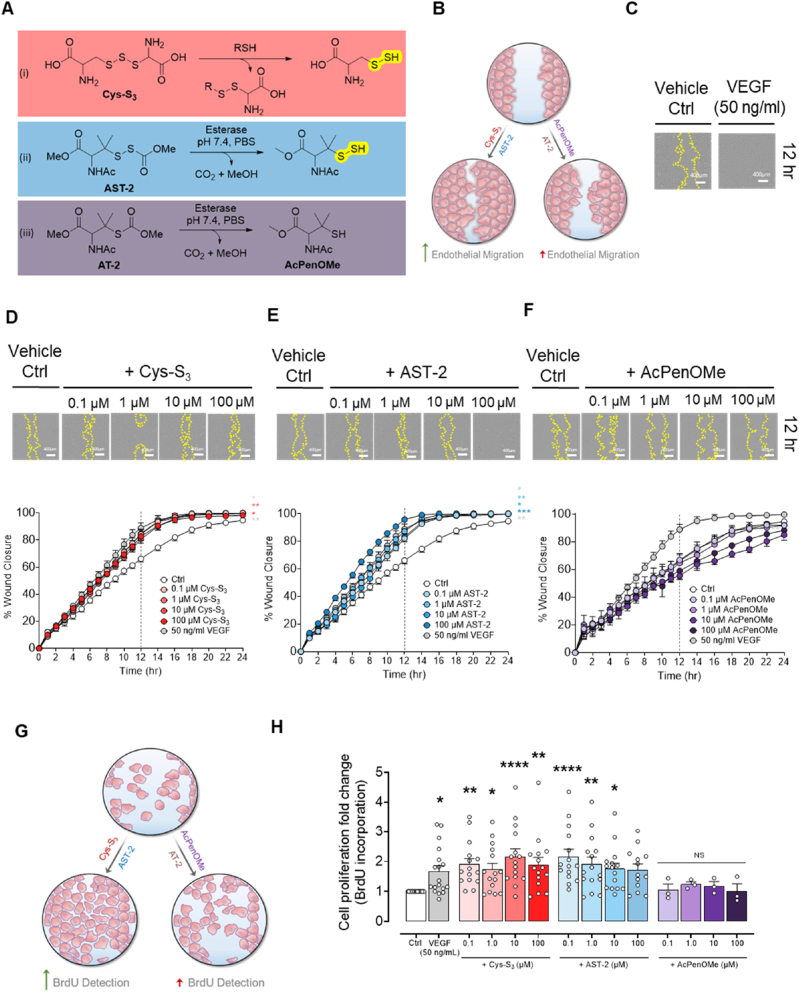
**Hydropersulfide donors promote migration rate and proliferation in human umbilical vein endothelial cells A,** Reaction scheme showing RSSH and RSSH release from RSSH donors (Cys-S_3_ and AST-2) and thiol control (AT-2). **B,** Schematic overview of the scratch wound assay for RSSH donors (Cys-S_3_ and AST-2) and thiol controls (AT-2 and AcPenOMe (*N*-acetyl-penicillamine *O*-methyl ester)). **C–F,** Serum-starved HUVECs were scratch-wounded from 3 independent HUVEC donors and treated with either (**C**) vehicle control (Ctrl; n = 12), VEGF (50 ng/ml; n = 12), or increasing concentrations (0.1, 1, 10, or 100 μM) of (**D**) Cys-S_3_ (Cys-SSH donor, n = 6), (**E**)**,** AST-2 (*N*-acetyl *O*-methyl penicillamine hydropersulfide donor, n = 6), or (**F**), *N*-acetyl-penicillamine *O*-methyl ester (AcPenOMe, thiol control, n = 4). Wound closure was monitored over 24 h using IncuCyte. Both Cys-S_3_ and AST-2 promoted migration similarly to VEGF, while AcPenOMe showed no effect. Representative wound images shown at 12 h post-treatment. **G**, Serum-starved HUVECs were treated with either vehicle control (n = 18), AcPenOMe (n = 3), or RSSH donors (Cys-S_3_ and AST-2; n = 15) for 24 h and proliferation was measured by BrdU assay. **H**, Both Cys-S_3_ AST-2 induced concentration-dependent increases in cell proliferation, comparable to the effect of VEGF (n = 18). **B**–**F** were analysed by two-way ANOVA and **H**, one-way ANOVA with Dunnett's post-hoc test. Data presented as mean ± SEM; **D-F**, ∗P < 0.05; ∗∗P < 0.01; ∗∗∗P < 0.001 vs control. **H**, ∗P < 0.05; ∗∗P < 0.01, ∗∗∗∗P < 0.0001 *vs* control.

We employed two mechanistically distinct RSSH-releasing agents, cysteine trisulfide (Cys-S_3_) and alkylsulfenyl thiocarbonate-2 (AST-2), to investigate the role of RSSH in the regulation of angiogenesis. Cys-S_3_ undergoes rapid thiol–disulfide exchange with endogenous thiols (RSH) such as GSH and Cys-SH, producing Cys-SSH and an asymmetrical disulfide byproduct (Fig. 1A, Eq. i) [22]. As previously demonstrated, this precursor efficiently elevates intracellular RSSH levels [31]. In contrast, AST-2 is an esterase-activated precursor that releases *N*-acetyl penicillamine methyl ester hydropersulfide (AcPenOMe-SSH) with ca. 95% efficiency (*t*_1⁄2_ = 50.1 ± 1.8 min in pH 7.4 PBS at 37 °C in the presence of porcine liver esterase (PLE; Fig. 1A, Eq. ii; [32]). These donors offer complementary activation mechanisms: Cys-S_3_ is responsive to cellular thiols, while AST-2 is activated by an esterase enzyme, enabling control over intracellular RSSH release. Notably, the chemical identity of the released RSSH differs - Cys-S_3_ generates the endogenous Cys-SSH, whereas AST-2 liberates a sterically hindered *t-*alkyl hydropersulfide that exhibits greater stability and superior reactivity in transpersulfidation reactions, i.e., sulfane sulfur transfer to cellular thiols, thereby generating corresponding RSSH species [33]. To ascertain the observed effects are due to RSSH, we synthesized a thiol control alkyl thiocarbonate-2 (AT-2), an analog of AST-2, that slowly releases *N*-acetyl-penicillamine methyl ester (AcPenOMe) upon esterase activation but does not generate RSSH (Fig. 1A, Eq.iii). AcPenOMe itself was also used as an additional control to test the effects of the thiol alone (Fig. 1A, Eq.iii).

### 3.2 Exogenously produced RSSH induces angiogenesis in HUVECs

In the present study, we first tested whether these RSSH donors promote angiogenesis *in vitro* by measuring HUVEC migration using a scratch wound assay (Fig. 1B). Migration dynamics were compared to VEGF, a well-known proangiogenic mediator (Fig. 1C). Treatment with Cys-S_3_ consistently increased cell migration uniformly across all pharmacological concentrations tested (Fig. 1D). In contrast, AST-2 induced a concentration-dependent increase in migration, with the highest concentration producing a response similar in both time-course and magnitude to the proangiogenic effect of VEGF (Fig. 1E). Building on the novel observation that exogenous RSSH treatment promotes scratch wound closure, we further validated this observation using structurally related control thiol compounds to confirm that the pro-angiogenic responses were specifically attributable to RSSH. Neither AcPenOMe nor AT-2 (Fig. 1F and Supplementary Fig. 2A) promoted scratch wound closure. In addition to migration, increased cell proliferation is essential for vessel growth. We therefore tested whether RSSH donors affect HUVEC cell proliferation (Fig. 1G). Exogenous treatment with either Cys-S_3_ or AST-2 significantly increased endothelial cell proliferation (Fig. 1H) while maintaining viability across all pharmacologically relevant concentrations (Supplementary Figure 2B). In contrast, neither AcPenOMe nor AT-2 induced proliferation, but did sustain cell viability (Fig. 1H and Supplementary Fig. 2B and C, respectively).

**Fig. 2:**
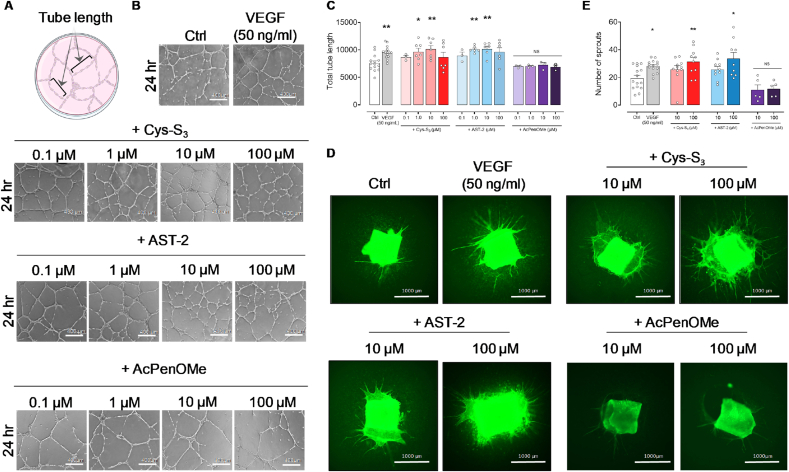
**Hydropersulfide donors potentiate *in vitro* endothelial tubulogenesis in HUVECs and *ex vivo* aortic sprouting**. **A**, Simplified schematic image for the assessment of HUVEC tube length. **B**, Representative images of tube networks of HUVECs treated with vehicle control (Ctrl), VEGF (50 ng/ml), and different concentrations (0.1, 1, 10, or 100 μM) of Cys-S_3_, AST-2 and AcPenOMe. Sub-confluent HUVECs from 3 independent donors were seeded onto Geltrex-coated wells and were treated with Ctrl (n = 14), VEGF (n = 14), Cys-S_3_ (n = 3-7), AST-2 (n = 3-6) or AcPenOMe (n = 3) for 24 h. Tube formation was assessed using an inverted phase contrast microscope (4x magnification). **C**, Quantitative analysis of total tube length showing Cys-S_3_ (1 and 10 μM) and AST-2 (1 and 10 μM) significantly increased total tube length compared to vehicle control (Ctrl), while AcPenOMe (thiol control) did not promote tubule formation in HUVECs. Data are presented as mean ± SEM. Statistical analysis was performed using one-way ANOVA followed by Dunnett's multiple comparison test, ∗p < 0.05, ∗∗p < 0.01 vs Ctrl. **D,** Aortic rings from C57BL/6 mice were embedded in rat-tail collagen-1 gel and cultured in Opti-MEM with either control medium (Ctrl), VEGF (50 ng/ml), Cys-S_3_, AST-2 or AcPenOMe with 10 or 100 μM for 7-days (n = 5-15 animals). Sprouting was assessed after staining endothelial cells with FITC-Lectin (Bandeiraea simplifolia) and imaged using Cell Discoverer 7 (10× magnification). **E**, Quantitative analysis of aortic sprouting. 100 μM of Cys-S_3_ and AST-2 significantly increased sprouting at similar extent as VEGF. Sprout counts were performed by two independent, blinded analysts. Data are shown as mean ± SEM. Statistical analysis used one-way ANOVA followed by Dunnett's post-hoc test, ∗P < 0.05; ∗∗P < 0.01 *vs* Ctrl.

Next, we examined the tubulogenic activity of RSSH donors using an *in vitro* tube formation assay (Fig. 2A). Both Cys-S_3_ and AST-2 significantly increased tube length compared to controls (vehicle control; Fig. 2B and C). Consistent with these *in vitro* HUVEC results, pharmacological concentrations of Cys-S_3_ and AST-2 also significantly enhanced vessel sprouting in *ex vivo* aortic ring assays (Fig. 2D). In contrast, the thiol controls (AcPenOMe and AT-2) showed minimal vessel growth in both HUVEC tube formation and aortic ring assays (Fig. 2B–E and Supplementary Fig. 3A–D, respectively). These findings support the pro-angiogenic role of exogenous RSSH on human endothelial cells and murine vascular tissue.

### 3.3 Endogenous RSSH produced via the CARS2/CPERS pathway promotes vessel growth

**Fig. 3:**
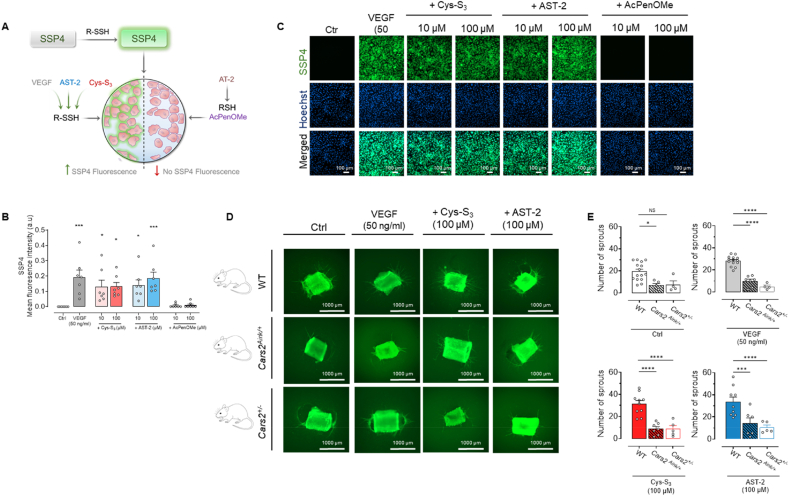
**VEGF and hydropersulfide donors induce polysulfides and sulfane-sulfur species in HUVECs and CARS2/CPERS is required for VEGF-driven aortic sprouting**. **A**, Schematic overview of SSP4 staining with RSSH donors and thiol controls. HUVECs were serum-starved for 4 h and then treated for 24 h with vehicle control (Ctrl), VEGF (20 or 50 ng/ml), Cys-S_3_, AST-2 or AcPenOMe (10 or 100 μM). Cells were fixed with 4% paraformaldehyde and stained with 20 μM SSP4 sulfane sulfur detection dye (green; detects persulfides and polysulfide derived species), Hoechst (200 μg/ml; blue, nuclear staining), and 150 μM CTAB (cetrimonium bromide) for 30 min. **B**, Quantification of SSP4 fluorescence intensity. Data are shown as mean ± SEM (n = 7/group; from 4 independent HUVEC donors). Statistical significance was determined by one-way ANOVA followed by Dunnett's post-hoc test; ∗p < 0.05, ∗∗∗p < 0.001 vs control. **C**, Representative fluorescence images showing SSP4 and Hoechst staining. **D**, Aortic rings from WT, *Cars2*^*+/−*^, and *Cars2*^*Aink/+*^ mice were embedded in rat-tail collagen-1 gel and cultured in Opti-MEM with either control medium (Ctrl), VEGF (50 ng/ml), Cys-S_3_, AST-2 or AcPenOMe (10 or 100 μM) for 7-days. Sprouting was assessed after staining endothelial cells with FITC-Lectin (Bandeiraea simplifolia) and imaged using a Zeiss Cell Discoverer 7 (10× magnification). **E**, VEGF or RSSH donors did not promote sprouting in the *Cars2*^*+/−*^ and *Cars2*^*Aink/+*^ aortic rings when compared to the WT mice. Sprout counts were performed by two independent, blinded analysts. Data are shown as mean ± SEM from n = 4-15 animals. Statistical analysis used one-way ANOVA followed by Sidak's post-hoc test, ∗P < 0.05; ∗∗∗P < 0.001, ∗∗∗∗P < 0.0001 *vs* control (Ctrl).

Previous studies have demonstrated that VEGF stimulates H_2_S production [4,6]. Building on this, we hypothesised that endogenous RSSH is essential for VEGF-driven angiogenic signalling. Using a specific sulfane-sulfur fluorescent probe, SSP4 [26] (Fig. 3A), we observed increased RSSH levels following VEGF stimulation, with a marked elevation at 24 h exposure compared to 1 h treatment (Fig. 3B and C, and Supplementary Fig. 4A and B, respectively). As expected, RSSH donors but not the thiol control increased sulfane sulfur levels in HUVECs. Furthermore, we validated the SSP4 findings using a more sensitive and quantitative LC-MS/MS employing sulfane sulfur trapping by triphenylphosphine to detect polysulfides (sulfane sulfur). This analysis further supports that VEGF and RSSH donors increase the formation of polysulfide species in HUVECs [Supplementary Figure 4C].

To evaluate the functional importance of VEGF-induced RSSH in angiogenesis, we employed RSSH-deficient mice, *Cars2*^*+/−*^ and *Cars2*^*Aink/+*^, both heterozygous for mitochondrial cysteinyl-tRNA synthetase (*CARS2*). As previously described, RSSH reduction is milder in *Cars2*^*AINK/+*^ mice, where only one of the catalytic sites for RSSH biosynthesis is disrupted, whereas *Cars2*^*+/−*^ mice lack both sites, resulting in a more substantial decrease in RSSH production [13,34]. In the present study, VEGF-induced aortic sprouting was markedly reduced in *Cars2*^*+/−*^ mice compared to both *Cars2*^*Aink/+*^ and their WT littermates (Fig. 3D and E). Furthermore, treatment with RSSH donors, Cys-S_3_ or AST-2, failed to rescue aortic sprouting in either *Cars2*^*Aink/+*^ or *Cars2*^*+/−*^ mice (Fig. 3D and E).

### 3.4 RSSH-induced angiogenesis is mediated by the Akt-eNOS signalling axis and acts through the NO-cGMP pathway

**Fig. 4:**
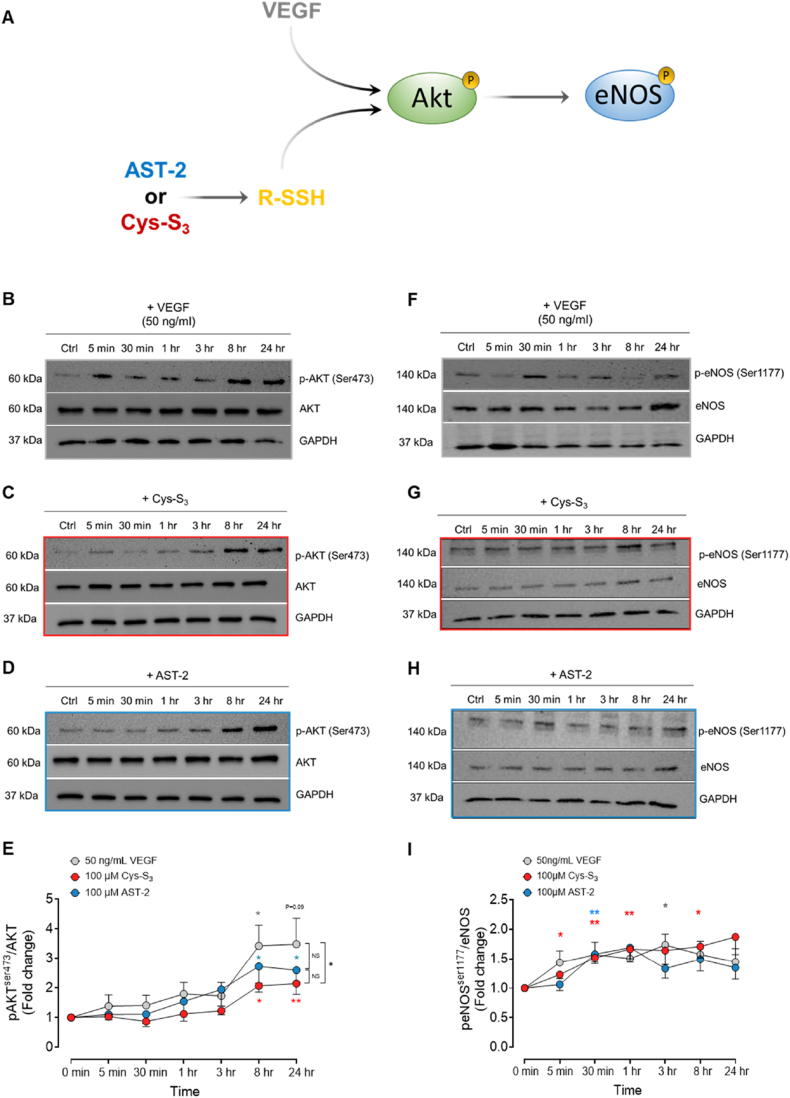
**Hydropersulfide stimulates phosphorylation of Akt and eNOS pathway.** (**A**) HUVECs were serum deprived overnight and then treated with vehicle control (Ctrl), VEGF (50 ng/ml), Cys-S_3_ or AST-2 for 5 min, 30 min, 1 h, 3 h, 8 h or 24 h. Cell lysates were prepared and analysed by SDS/PAGE. PVDF membranes were probed with antibodies specific for the phosphorylated and total forms of (**B-E**) pAkt^ser473^ and Akt (**F-I**) peNOS^ser1177^ and eNOS by Western blotting. Blots shown are representative from 8 to 11 different experiments from 4 independent HUVEC donors. Data shown as mean ± SEM. **B–I**, Statistical significance was determined by two-way ANOVA with Sidak's post hoc test. ∗P < 0.05 Cys-S_3_ vs VEGF; P > 0.05 AST-2 vs VEGF or Cys-S_3_ (non-significant; NS); one-way ANOVA with Dunnett's post hoc test ∗P < 0.05 vs Ctrl; ∗∗P < 0.01 vs Ctrl.

**Fig. 5:**
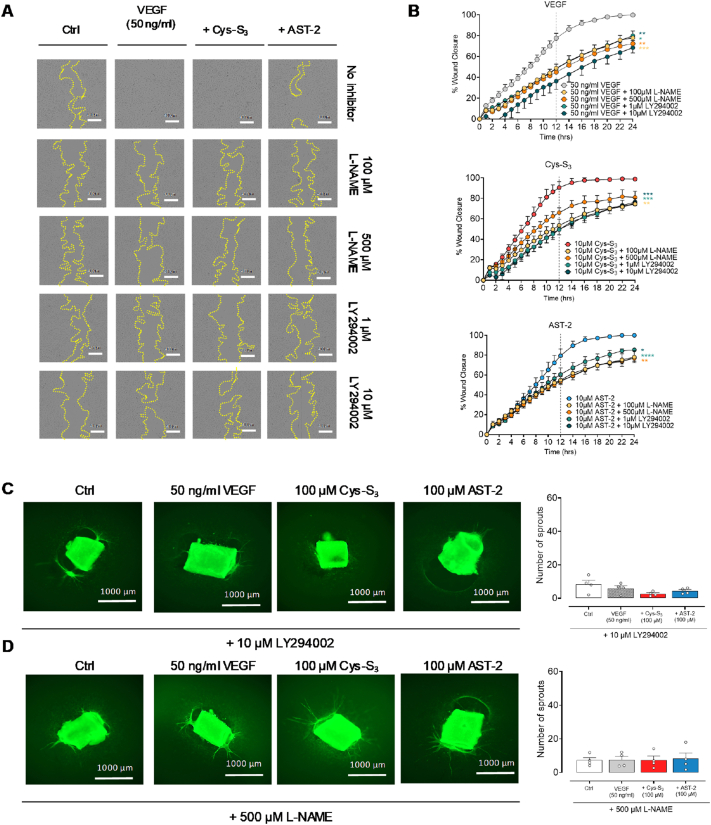
**Akt and NOS inhibition prevents hydropersulfide-induced angiogenesis**. HUVECs were serum-starved overnight, then pre-treated for 30 min with either l-NAME (100 or 500 μM) or LY294002 (1 or 10 μM) to inhibit eNOS or Akt, respectively. After pre-treatment, cells were incubated with vehicle control (Ctrl), VEGF (50 ng/ml), Cys-S_3_ (10 μM) or AST-2 (10 μM) for 24 h. Wound closure was monitored using IncuCyte over a 24 h period. **A**, Representative wound images at 12 h post-treatment. **B**, Both l-NAME and LY294002 significantly reduced Cys-S_3_ and AST-2-induced migration, comparable to the reduction seen with VEGF treatment. Data are shown as mean ± SEM from n = 5 experiments from 3 independent HUVEC donors. Statistical significance was assessed by two-way ANOVA followed by Dunnett's post hoc test. ∗P < 0.01, ∗∗P < 0.05, ∗∗∗P < 0.001, ∗∗∗∗P < 0.0001 vs treatment in the absence of inhibitors (l-NAME or LY294002). Representative images of aortic rings from C57BL/6 mice that were embedded in rat-tail collagen-1 gel and cultured in Opti-MEM with either (**C**) Akt inhibitor (10 μM LY294002) or (**D**) NOS inhibitor (500 μM l-NAME) was added for 30 min, followed by control medium (Ctrl), VEGF (50 ng/ml), 100 μM Cys-S_3_, or 100 μM AST-2 for 7-days (n = 4/group animals). Sprouting was assessed after staining endothelial cells with FITC-Lectin (Bandeiraea simplifolia) and imaged using Cell Discoverer 7 (10× magnification). **C-D**, Quantitative analysis of aortic sprouting. Inhibition of Akt and NOS prevented Cys-S_3_ and AST-2-induced sprouting to similar extent as VEGF. Sprouts counts were performed by two independent, blinded analysts. Data are shown as mean ± SEM. Statistical analysis used one-way ANOVA followed by Dunnett's post-hoc test, P > 0.05 *vs* control (Ctrl).

Having confirmed that both endogenous and exogenous RSSH exhibit pro-angiogenic properties, we next sought to understand how RSSH triggers angiogenic signalling. We focussed on the classical canonical VEGF-induced signalling through phospho-activation of the Akt-eNOS pathway [35]. HUVECs were serum-starved overnight, then treated with VEGF, Cys-S_3_ or AST-2. We assessed total and phosphorylated levels of Akt and eNOS over a 24h time course (Fig. 4A). Both Cys-S_3_ and AST-2 transiently increased phosphorylation of Akt at Ser473 and eNOS at Ser1177 (Fig. 4B–I, respectively). While inhibition of Akt (with LY294002) or NOS activity (with l-NAME) significantly impaired HUVEC migration in the scratch wound healing assay (Fig. 5A and B, and Supplementary Fig. 5A and 5B), as well as aortic ring sprouting in response to Cys-S_3_ or AST-2 (Fig. 5C and D). Inhibitory activity was validated by Western blotting (Supplementary Figure 6).

To determine whether the pro-angiogenic effects of Cys-S_3_ and AST-2 are mediated through activation of the NO-sGC-cGMP-PKG-VASP pathway, we next measured intracellular cGMP levels and the phosphorylation of VASP at serine 239, a well-established marker of protein kinase G (PKG) activity. Both Cys-S_3_ and AST-2 significantly enhanced NO-induced cGMP accumulation and VASP^ser239^ phosphorylation compared with the NO donor SPER-NO alone (Supplementary Fig. 7A and 7B, respectively).

### 3.5 CARS2/CPERS pathway is required for nitric oxide-mediated vasorelaxation

**Fig. 6:**
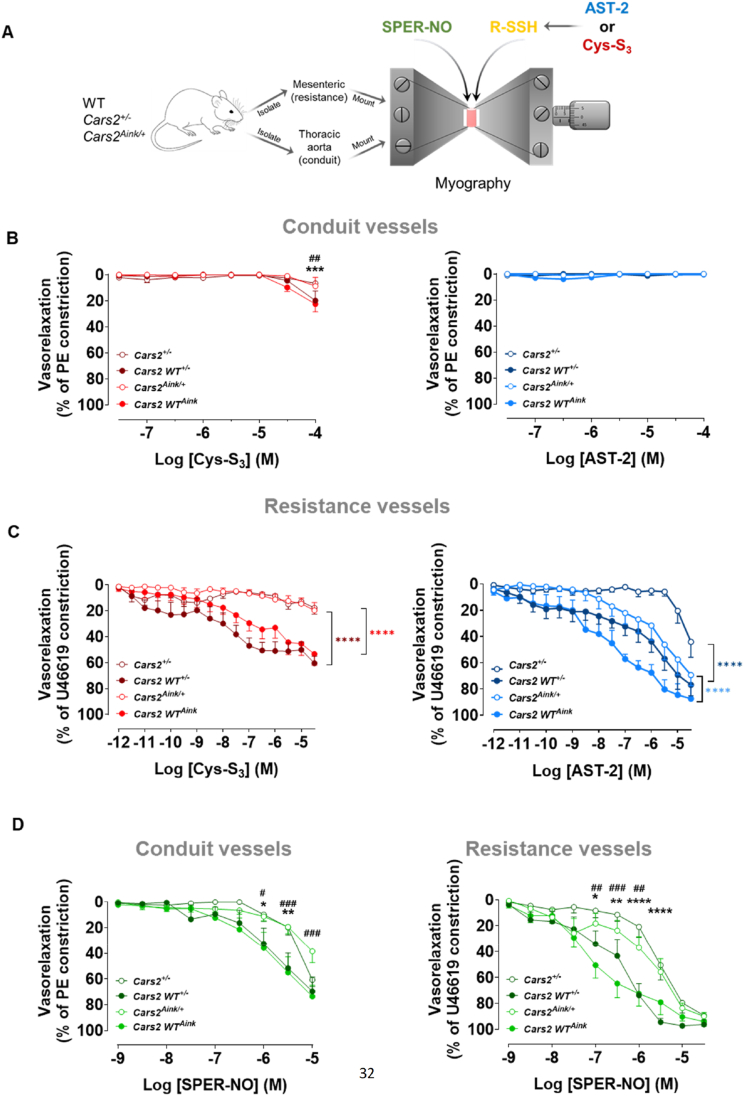
**Hydropersulfide and NO requires CARS2/CPERS to mediate vasorelaxation**. **A**, Schematic of the experimental design to assess *ex vivo* vasorelaxation responses to RSSH (Cys-S_3_ and AST-2) and NO (spermine-NONOate; SPER-NO) donors in isolated mesenteric (resistance) and thoracic aorta (conduit) blood vessels from Cars2^+/−,^*Cars2*^Aink/+^ and their wild-type (WT) littermates. Vessels were mounted in a myograph and pre-constricted with U46619 (mesenteric arteries) and phenylephrine (thoracic aorta). Concentration-response curves to Cys-S_3_, AST-2 and SPER-NO were constructed. **B**, Cys-S_3_ and AST-2 produced less vasodilation in aortic rings from both *Cars2*^*+/−*^, *Cars2*^*Aink/+*^ and their wild-type (WT) littermates. **C**, Relaxation to Cys-S_3_ and AST-2 was significantly reduced in both mesenteric arteries from *Cars2*^*+/−*^ and *Cars2*^*Aink/+*^ when compared with WT. **D**, NO-induced relaxation was significantly attenuated in both thoracic aorta and mesenteric resistance arteries from *Cars2*^*+/−*^ and *Cars2*^*Aink/+*^ when compared with WT. **B-D,** Data are presented as mean ± SEM (n = 5-9 animals). Significance between curves was assessed using (**B**) 2-way ANOVA followed by Šídák's comparison test ∗∗∗P < 0.0001 compared with *Cars2*^*+/−*^ vs *Cars2* WT^+/−^; ^##^P < 0.001 compared with *Cars2*^*Aink/+*^ vs *Cars2 WT*^*Aink*^. (**C**) 2-way ANOVA followed by Šídák's comparison test ∗∗∗∗P < 0.00001 compared with *Cars2*^*+/−*^ vs *Cars2* WT ^±^ and *Cars2*^*Aink/+*^ vs *Cars2 WT*^*Aink*^. (**D**) 2-way ANOVA followed by Tukey's test ∗P < 0.05, ∗∗P < 0.01, ∗∗∗∗P < 0.00001 compared with *Cars2*^*+/−*^ vs *Cars2* WT ^±^ and ^#^P < 0.05, ^##^P < 0.01, ^###^P < 0.001 compared with *Cars2*^*Aink/+*^ vs *Cars2 WT*^*Aink*^.

Based on current evidence that RSSH require the eNOS-NO signalling pathway for angiogenesis, and given the established role of H_2_S–NO interactions in both angiogenesis and vasorelaxation [6], we investigated whether the RSSH/CARS2/CPERS pathway is essential for vascular tone, and whether NO depends on this pathway in conduit (thoracic aorta) or resistance vessels – a question that has remained unanswered up to now. To this end, we constructed cumulative concentration-response curves to RSSH donors, Cys-S_3_ and AST-2 (0.1 nM-100 μM), in isolated pre-constricted thoracic aorta and mesenteric resistance vessels from *Cars2*^*Aink/+*^ and *Cars2*^*+/−*^ mice and their WT littermates (Fig. 6A). Both Cys-S_3_ and AST-2 failed to induce relaxation in isolated aortic rings from any mouse strain, although Cys-S_3_ produced a slight potentiation at 100 μM (Fig. 6B). In contrast, RSSH donors elicited concentration-dependent relaxation in WT resistance vessels but showed significantly attenuated vasorelaxant effects in *Cars2*^*Aink/+*^ and *Cars2*^*+/−*^ resistance vessels (Fig. 6C).

To determine whether NO-mediated vasorelaxation depends on an RSSH-induced CARS2/CPERS pathway, we constructed cumulative concentration-response curves to an NO donor, spermine NONOate (SPER-NO), in isolated pre-constricted thoracic aorta and mesenteric resistance vessels from *Cars2*^*Aink/+*^ and *Cars2*^*+/−*^ mice and their WT littermates (Fig. 6D). SPER-NO (0.1μM-1 mM) relaxed both aortic and mesenteric blood vessels from WT mice in a concentration-dependent manner (Fig. 6D). However, relaxation to SPER-NO was significantly impaired in vessels from *Cars2*^*Aink/+*^ and *Cars2*^*+/−*^ mice compared with their WT controls (Fig. 6D), indicating that NO requires the CARS2/CPERS pathway to dilate both conduit and resistance vessels.

## 4 Discussion

RSSH are recognized for their potent antioxidant and cytoprotective properties in mammalian cells [9,15,36]. While these properties suggest a physiological role for RSSH, their contribution to the regulation of angiogenesis has remained unknown. Recent studies using polymeric RSSH delivery systems demonstrated proangiogenic effects [19,20], but they did not offer mechanistic insight and controlled, donor-specific evaluation across multiple angiogenesis models. In this study, we show that exogenous administration of RSSH promotes endothelial cell migration, proliferation, and aortic sprouting via action of the Akt-eNOS and NO-cGMP pathway. Conversely, using two distinct Cys-SSH producing enzyme CARS2/CPERS mutant mouse models, we demonstrate that RSSH-driven angiogenesis is assisted by the CARS2/CPERS- mechanism and plays a role in blood vessel formation. Notably, the vasodilatory response to NO donor, SPER-NO, was attenuated in these mutants, indicating that effective NO signalling in conduit and resistance vessels requires an intact CARS2/CPERS pathway. Another observation from our study is that VEGF stimulation appears to induce endogenous RSSH production, thereby facilitating angiogenesis. Collectively, these findings suggest a potential role for RSSH in regulating angiogenesis and vascular tone.

H_2_S-based therapeutics have gained traction over the past decade as treatments for promoting angiogenesis [37,38]. However, their molecular mechanisms remain poorly understood. Recent studies suggest that some biological effects previously attributed to H_2_S may instead result from its oxidized congeners - namely RSSH and RSS_*n*_R [9]. RSSH can form via both H_2_S-dependent and H_2_S- independent routes [39]. In H_2_S-dependent pathways, H_2_S reacts with oxidized thiols such as Cys-SH and GSH to produce Cys-SSH and GSSH [7,10,14]. It can also react with disulfides (RSSR) or sulfenic acids (RSOH) to yield the corresponding RSSH [39]. Additionally, H_2_S can add to the trisulfide cofactor of coenzyme A (CoA) to form CoA-SSH [8]. In contrast, H_2_S-independent formation occurs through reactions involving oxidants such as hydroxyl (HO^•^), carbonate (CO_3_^•-^) and nitrogen dioxide (NO_2_^•^) radicals, or between sulfur compounds with thiols [39]. Furthermore, RSSH can readily modify protein cysteine residues (P-SH), leading to the formation of protein hydropersulfides (P-SSH) [40]. Despite these promising insights, studying the biological functions of RSSH remains challenging due to their intrinsic chemical instability and the limited availability of well-characterised donor compounds.

Consequently, understanding their precise role in cardiovascular homeostasis is still in its early stages. To address this, we developed novel donor compounds to clarify whether RSSH act as direct pro-angiogenic mediators. In this study, we employed two distinct donors, Cys-S_3_ and AST-2, to modulate RSSH levels. Our results demonstrate that incubation of HUVECs with these RSSH donors promotes endothelial cell migration and proliferation, mirroring the combined effects of H_2_S and NO reported in previous studies [6]. These findings suggest that RSSH donors may have therapeutic potential as modulators of angiogenesis and enhancers of wound healing.

VEGF is known to stimulate the production of both H_2_S and NO, which cooperatively interact to regulate angiogenesis [4,6]. Intriguingly, we found that VEGF also promotes endogenous RSSH and related polysulfides production in the HUVECs. This increase was detected using the SSP4 fluorescent probe and further confirmed by a sensitive and quantitative LC-MS/MS analysis. Previously, it has been shown that mitochondrial CARS2 serves as the primary cysteine persulfide synthase (CPERS), directly generating Cys-SSH. Additionally, both *Cars2*^+/−^ and *Cars2*^*Aink/+*^ mice exhibit significantly reduced levels of various RSSH-containing metabolites, including Cys-SSH, GSSH, HSSH, and HSSSH [13]. However, whether CARS2/CPERS plays a functional role in VEGF-induced angiogenesis remained unclear. In the present study, we demonstrate a previously unrecognized role for CARS2/CPERS-dependent RSSH production in promoting angiogenic activity. Our results indicate that impaired CARS2/CPERS – as observed in *Cars2*^+/−^ and *Cars2*^*Aink/+*^ mice – appears to attenuate VEGF-induced aortic sprouting. However, the involvement of specific VEGF signalling components has not been directly examined in this study. Interestingly, similar effects were observed when CARS2 deficient aortic rings were pharmacologically treated with RSSH donors. This unexpected finding suggests that further investigation is needed to compare aortic tissue with other vascular beds and their corresponding WT controls, to clarify the VEGF signalling mechanisms involved in CARS2-deficiency. Overall, while the present study demonstrates altered angiogenic response, future work employing endothelial cells derived from *Cars2*^+/−^ and *Cars2*^*Aink/+*^ would be valuable to directly assess the cellular mechanisms underlying the observed genotype-dependent effects.

A major feature of RSSH in chemical biology, which is distinct from thiols, is their dual reactivity. Depending on their protonation state, RSSH can act as either nucleophiles or electrophiles [36]. This enables them to engage in a range of redox reactions with electrophiles, including reactive oxygen species like hydrogen peroxide, and participate in redox signalling by modifying cysteine residues on target proteins to generate P-SSH [40]. Given this adaptability, RSSH may play a central role in redox-mediated signalling networks and the underlying pro-angiogenic effects are likely complex and remain incompletely defined. Because previous evidence supports the link between H_2_S and eNOS–NO–sGC-cGMP signalling with angiogenic and vasorelaxant responses [6,38]. We investigated a well-characterised pathway known to regulate vascular homeostasis and growth, the Akt-eNOS signalling axis [41]. We found that exogenous RSSH increased HUVEC phosphorylation of Akt^ser473^ and eNOS^ser1177^. Moreover, pharmacological inhibition of Akt and NOS significantly attenuated both scratch closure and sprouting responses to RSSH donors, confirming that Akt-eNOS activation is a key component of this pro-angiogenic signalling pathway. Furthermore, both RSSH donors, Cys-S_3_ and AST-2, increased NO-stimulated intracellular cGMP accumulation and activated the PKG downstream effector VASP^ser239^ in HUVECs. However, it is plausible that RSSH promotes angiogenesis through additional pro-angiogenic signalling pathways. H_2_S has been shown to enhance eNOS activity by inducing S-sulfhydration, a modification that promotes eNOS phosphorylation and dimerization, thereby increasing its activity and elevating NO bioavailability [42]. Beyond the canonical NO-sGC-cGMP-PKG pathway, we and others have demonstrated that RSSH [14] and polysulfur species derived from H_2_S [43] can activate protein kinase G (PKG) independently of this pathway by oxidizing the cysteine-42 residue on PKG1α, which induces vasodilation in the resistance vessels and blood pressure lowering effect [14,43]. These findings are further supported by the observation that the Cys-S_3_ donor can dimerize PKG1α [44]. Therefore, additional investigations are warranted to elucidate the significance of these redox-mediated pathways and other potential posttranslational modifications in angiogenesis and other cardiovascular disease models [[45], [46], [47]].

Having established the potential importance of RSSH and the CARS2/CPERS pathway in angiogenesis, we next examined their role in vascular tone regulation. Our data show that the CARS2/CPERS pathway contributes to RSSH-mediated vasorelaxation in resistance vessels, but not in conduit vessels. Notably, NO-induced vasorelaxation occurred in both conduit and resistance vessels in WT mice; however, this response was significantly attenuated in *Cars2*^+/−^ and *Cars2*^*Aink/+*^ mice, indicating that NO also depends on the CARS2/CPERS pathway in both vessel types.

These findings reveal a role for the CARS2/CPERS pathway in mediating vasorelaxation to both RSSH and NO, with RSSH showing vascular bed-specific effects. In the present study, we found that CARS2 deficient mice were unresponsive to VEGF, and RSSH treatment failed to rescue this effect in aortic rings. Taken together, these results suggest that the physiological effects of RSSH are complex and tissue specific, reflecting distinct regulatory mechanisms in resistance versus conduit vessels. Further investigation is needed to determine the factors underlying these differences and which RSS species – RSSH metabolites, CoA-SSH, P-SSH or H_2_S directly contributes to angiogenesis and vascular tone. Furthermore, because RSSH donors are highly reactive, their biological effects likely go beyond direct signalling. They may also drive broader changes in the cellular S-persulfidation landscape, as recently reported by Wittig and colleagues [48]. In addition, RSSH may undergo further metabolism to generate H_2_S or elemental sulfur. Therefore, future studies examining the expression and activity of other key H_2_S metabolizing enzymes such as CSE, CBS and 3-MST after prolonged RSSH exposure would be valuable for elucidating the downstream metabolic fate and biological consequences of these sulfur species. Notably, our findings highlight the CARS2/CPERS pathway, which is the principal enzymatic source of Cys-SSH [13,49,50], as a potential therapeutic target for controlling angiogenesis and vascular tone.

In summary, our findings suggest that RSSH can drive angiogenesis through redox-sensitive mechanisms, with the CARS2/CPERS signalling axis as a potential regulatory component. These results demonstrate how RSSH species influence vascular development, highlighting redox modulation as a contributor in endothelial cell activation, blood vessel formation, and vascular tone. While these observations from our CARS2/CPERS-CysSSH deficient mouse model support a role for RSSH in vascular regulation, we acknowledge that RSSH donors may also give rise to H_2_S under cellular reducing conditions and that vessel remodelling under physiological conditions is also influenced by multiple compensatory pathways and enzymes, which may buffer the effects of CARS2 deficiency. Further studies are needed to specifically characterise the underlying redox mechanisms underpinning RSSH-driven angiogenesis and vasorelaxation, including more detailed dissection of VEGF-related signalling pathways and their regulation at the transcriptomic and protein levels. Such approaches, including targeted proteomic analysis or unbiased transcriptomic profiling, would help to define the downstream molecular networks involved. These studies will also be important to assess the broader therapeutic potential of modulating RSSH signalling in strategies aimed at promoting or inhibiting vascular growth in different disease scenarios. Overall, these insights lay the foundation for further studies into the role of RSSH in vascular biology and regulation.

## Funding

This work was supported by the 10.13039/501100000274British Heart Foundation (PG/23/11579) awarded to M. Madhani and the US National Science Foundation (CHE-2247616) to JPT. Grants-in-Aid for Scientific Research [Transformative Research Areas, Challenging Exploratory Research, International Leading Research, (S)] from the Ministry of Education, Culture, Sports, Science and Technology (MEXT), Japan, to T. Akaike (21H05263, 22K19397, 23K20040, 24H00063); by the Japan Science and Technology Agency (JST) CREST Grant Number JPMJCR2024 to T. Akaike; and by the Japan Agency for Medical Research and Development (AMED) Grant Number JP21zf0127001 to T. Akaike. The National Institute of 10.13039/100018696Health and Care Research (NIHR) 10.13039/501100018952Birmingham Biomedical Research Centre (NIHR203326) have supported the 10.13039/501100000855University of Birmingham Department of Cardiovascular Sciences where this research is based.

## CRediT authorship contribution statement

**Reece J. Lamb:** Data curation, Formal analysis, Writing – review & editing. **Fifi S. Ibrahim:** Data curation, Formal analysis, Writing – review & editing. **Vinayak S. Khodade:** Data curation, Formal analysis, Writing – review & editing. **Scott P. Davies:** Data curation, Formal analysis, Writing – review & editing. **Kayleigh Griffiths:** Data curation, Formal analysis, Writing – review & editing. **Tsuyoshi Takata:** Data curation, Formal analysis, Writing – review & editing. **Jinjing Gu:** Data curation, Formal analysis, Writing – review & editing. **Tetsuro Matsunaga:** Resources, Writing – review & editing. **Roger J. Grand:** Data curation, Formal analysis, Writing – review & editing. **Francesca M. Nichols:** Data curation, Formal analysis, Writing – review & editing. **Alice J. Barton:** Data curation, Formal analysis, Writing – review & editing. **Aisah A. Aubdool:** Methodology, Writing – review & editing. **Masanobu Morita:** Resources, Writing – review & editing. **Hozumi Motohashi:** Resources, Writing – review & editing. **Ming Xian:** Resources, Writing – review & editing. **Takaaki Akaike:** Funding acquisition, Resources, Writing – review & editing. **John P. Toscano:** Formal analysis, Funding acquisition, Resources, Validation, Writing – original draft, Writing – review & editing. **Melanie Madhani:** Conceptualization, Formal analysis, Funding acquisition, Investigation, Methodology, Project administration, Resources, Supervision, Validation, Writing – original draft, Writing – review & editing.

## Declaration of competing interest

The authors declare that they have no known competing financial interests or personal relationships that could have appeared to influence the work reported in this paper.

## Data Availability

Data will be made available on request.
