# Supplementary material for: Hydropersulfides promote angiogenesis and preserve vascular function
Source: Redox Biol. 2026 Apr 30;94:104192. doi: 10.1016/j.redox.2026.104192 (PMC13158569; doi:10.1016/j.redox.2026.104192)
Supplement: Multimedia component 1 [file mmc1.docx]

**Supplementary Information**

**Hydropersulfides Promote Angiogenesis and Preserve Vascular Function**

Reece J Lamb^1#^, Fifi S Ibrahim^1#^, Vinayak S Khodade^2#^, Scott P Davies^3^, Kayleigh Griffiths^1^, Tsuyoshi Takata^4^, Jinjing Gu^2^, Tetsuro Matsunaga^4^, Roger J Grand^1^, Francesca M Nichols^1^, Alice J Barton^1^, Aisah A Aubdool^5^, Masanobu Morita^4^, Hozumi Motohashi^6^, Ming Xian^7^, Taka Akaike^4^, John P Toscano^2#*^, Melanie Madhani^1,8 #*^

^1^Department of Cardiovascular Sciences, School of Medical Sciences, College of Medicine and Health, University of Birmingham, United Kingdom

^2^Department of Chemistry, Johns Hopkins University, Baltimore, MD, USA

^3^Deparment of Inflammation and Immunotherapy, School of Infection, Inflammation, and Immunology, College of Medicine and Health, University of Birmingham, United Kingdom

^4^Department of Redox Molecular Medicine, Tohoku University Graduate School of Medicine, Sendai, 980-8575, Japan.

^5^William Harvey Research Institute, Faculty of Medicine and Dentistry, Barts & The London Hospitals, Queen Mary University of London, United Kingdom

^6^Department of Medical Biochemistry, Tohoku University Graduate School of Medicine, Sendai 980-8575, Japan

^7^Department of Chemistry, Brown University, USA

^8^National Heart Research Institute Singapore, National Heart Centre Singapore

^#^These authors contributed equally

*Corresponding authors

Melanie Madhani John P Toscano

Department of Cardiovascular Sciences Department of Chemistry

College of Medicine and Health Johns Hopkins University

University of Birmingham Baltimore, MD 21218

B15 2TT, United Kingdom USA

Email address: [m.madhani@bham.ac.uk](mailto:m.madhani@bham.ac.uk) [jtoscano@jhu.edu](mailto:jtoscano@jhu.edu)

**Supplementary Figure 1. Cell Profiler enables accurate quantification of SSP4 fluorescence intensity in HUVECs.**

Flow diagram illustrating an example of Cell Profiler-segregation and discovery pipeline for detecting sulfane sulfur species within human umbilical endothelial vein endothelial cells (HUVECs). (1) Objects were generated by detecting individual HUVEC nuclei (Hoechst) and (2) sulfane sulfur probe 4 (SSP4) fluorescence. (3) The resultant objects were correlated using “relate objects” tool. At this stage, HUVEC that did not display SSP4 fluorescence at the set threshold were deleted. (4) Following this, the resulting objects were overlaid and subsequently categorised as HUVEC containing sulfane sulfur species, (5) these objects were subject to quantification by measuring mean SSP4 fluorescence intensity.

**Supplementary Figure 2. Thiol donor does not promote migration rate and proliferation in HUVECs**

**A**, Serum-starved HUVECs were scratch-wounded and treated with AT-2 (thiol control donor; n=4). Wound closure was monitored over 24 hr using IncuCyte and compared with vehicle control (Ctrl; n=12) and VEGF (50 ng/ml; n=12). AT-2 treatment had no effect on wound closure. Representative wound images are shown at 12 hr post-treatment. **B**, Trypan blue exclusion assay revealed no changes in cell viability from vehicle control, AcPenOMe, AT-2, Cys-S_3_ and AST-2 at all tested concentrations. Data are presented as mean ± SEM from 3 independent donors (n=3-6 per group). **C**, Serum-starved HUVECs were treated with control vehicle 50 ng/ml VEGF or AT-2 (0.1-100 μM) for 24 hr, and proliferation was assessed via BrdU assay. AT-2 did not induce cell proliferation compared to VEGF (n=15). **A** was analysed using two-way ANOVA and **B, C** one-way ANOVA with Dunnett’s post-hoc test **A,** **P<0.01 vs control; **B**, non-significant (NS) *vs* control; **C**, *P<0.05 vs control; P>0.05 (non-significant; NS) *vs* control.

**Supplementary Figure 3. Anti-angiogenic activity of thiol donor in endothelial and aortic sprouting *in vitro* models**

**A**, Representative images of tube networks of HUVECs following treatment with vehicle control (Ctrl), 50ng/ml VEGF, and AT-2 at various concentrations (0.1, 1, 10, or 100 μM) for 24 hr. Tube formation was assessed using an inverted phase contrast microscope (4x magnification) **B**, Quantitative analysis of total tube length showing AT-2 (n=3) did not promote tubule formation in HUVECs when compared to vehicle control (Ctrl; n=14) or VEGF (n=14). Data are presented as mean ± SEM from 3 HUVECs independent donors. Statistical analysis was performed using one-way ANOVA followed by Dunnett’s multiple comparison test, **P<0.05 vs Ctrl; P>0.05 vs Ctrl (NS; non-significant). **C,** Aortic rings from C57BL/6 mice were embedded in rat-tail collagen-1 gel and cultured in Opti-MEM with either control medium (Ctrl), VEGF (50ng/ml), or AT-2 with 10 or 100 μM for 7-days (n=8-14 animals). Sprouting was assessed after staining endothelial cells with FITC-Lectin (Bandeiraea simplifolia) and imaged using Cell discoverer 7 (10 x magnification). **D**, Quantitative analysis of aortic sprouting. AT-2 did not promote sprouting when compared to Ctrl or VEGF. Sprouts counts were performed by two independent, blinded analysts. Data are shown as mean ± SEM. Statistical analysis used one-way ANOVA followed by Dunnett’s post-hoc test, *P<0.05 *vs* control (Ctrl).

**Supplementary Figure 4. Absence of sulfane-sulfur production at early time point following VEGF and hydropersulfide exposure**

**A**, HUVECs were serum-starved for 4 hr and then treated for 1 hr with vehicle control (Ctrl), VEGF (50 ng/ml), Cys-S_3_, AST-2 or AcPenOMe (10 or 100 μM). Cells were fixed with 4% paraformaldehyde and stained with 20 µM SSP4 sulfane sulfur detection dye (green; detects RSSH and polysulfide-derived species), Hoechst (200 µg/ml; blue, nuclear staining), and 150 µM CTAB for 30 min. **B**, Quantification of SSP4 fluorescence intensity. Data are shown as mean ± SEM (n=4/group; from 3 independent HUVEC donors). Statistical analysis used one-way ANOVA followed by Dunnett’s post-hoc test; *p<0.05 vs control (Ctrl). **C**, HUVECs were serum-starved for 4 hr and then treated for 24 hr with vehicle control (Ctrl), VEGF (50 ng/ml), Cys-S_3_ or AST-2 (100 μM) followed by quantification of polysulfides and sulfane sulfur species with triphenylphosphine-based LC-MS/MS analysis. Data are shown as mean ± SEM (n=3/group; from 3 independent HUVEC donors). Statistical significance was determined by one-way ANOVA followed by Dunnett’s post-hoc test; **p<0.001, ****p<0.0001 vs control.

**Supplementary Figure 5. Validation that Akt and NOS inhibitor attenuate phosphorylation of Akt (Ser473) and eNOS (Ser1177) in the HUVECs.**

To examine the experimental conditions required to inhibit Akt and eNOS, HUVECs were serum-deprived overnight and pre-treated for 30 minutes with either LY294002 (1 or 10 μM) or L-NAME (100 or 500 μM). Cells were then stimulated with vehicle control (Ctrl) or VEGF (50 ng/ml) for 24 hr in the absence or presence of the inhibitors. Cell lysates were collected, resolved by SDS/PAGE, transferred to PVDF membranes. Western blotting was performed using antibodies against phosphorylated and total forms of (**A**) Akt (pAKT^ser473^ and AKT) and (**B**) eNOS (peNOS^ser1177^ and eNOS). Shown blots are representative examples of these conditions.

**Supplementary Figure 6. AKT and eNOS inhibition reduce Cys-S_3_ and AST-2-induced migration of HUVECs.**

HUVECs were serum-starved overnight, pre-treated for 30 minutes with the NOS inhibitor L-NAME (100 or 500 μM) or the AKT inhibitor LY294002 (1 or 10 μM), and then exposed to vehicle control (Ctrl), VEGF (50 ng/ml), Cys-S3 (100 μM) or AST2 (100 μM) for 24 hr. Wound closure was tracked by IncuCyte for 24 hr. **A**, Representative wound images at 12 hr post-treatment. **B**, Both L-NAME and LY294002 significantly suppressed Cys-S_3_ and AST-2-induced migration, to a degree comparable with VEGF treatment. Data are shown as mean ± SEM from n=3-5 experiments using 3 independent HUVEC donors. Statistical significance was assessed by two-way ANOVA followed by Dunnett’s post hoc test. *P<0.01, **P<0.05, ***P<0.001 vs corresponding treatment without inhibitor.

**Supplementary Figure 7. Hydropersulfide donors induce cGMP production and activate PKG-dependent phosphorylation VASP at ser239.**

**(A)** Following serum-starvation, HUVECs were re-suspended in endothelial cell growth medium containing IBMX (1mM) for 30 mins, then treated with vehicle control (Ctrl), VEGF (50 ng/ml), Cys-S_3_ (100μM), AST-2 (100μM), or the NO donor SPER-NO (100μM) for 1 hr. Intracellular cGMP levels were measured from 5-6 experiments using 4 independent HUVEC donors. **(B)** HUVECs were serum deprived overnight and treated with vehicle control (Ctrl), VEGF (50 ng/ml), Cys-S_3_ (100μM), AST-2 (100μM) or SPER-NO (100μM) for 1 hr. Cell lysates were prepared and analysed by SDS/PAGE. PVDF membranes were probed with antibodies against phosphorylated VASP (ser239) and total VASP. Blots shown are representative of 3 independent experiments using 3 different HUVEC donors. **A, B** Data shown as mean ± SEM. Statistical significance was determined by one-way ANOVA with Dunnett’s post hoc test. *P<0.05 or **P<0.01 vs Ctrl.


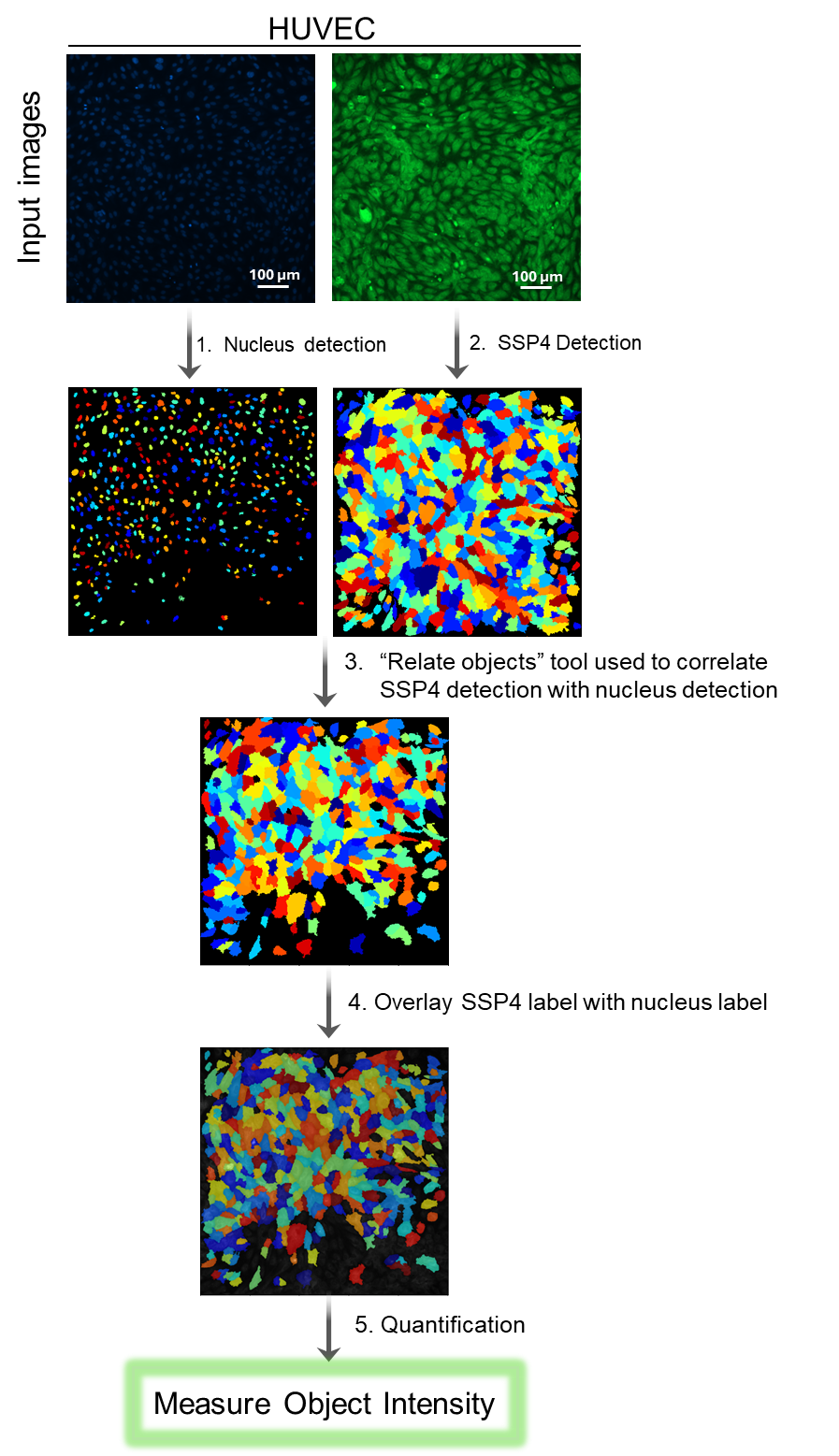

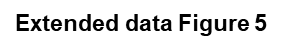


**Supplementary Figure 1**

**Supplementary Figure 2**


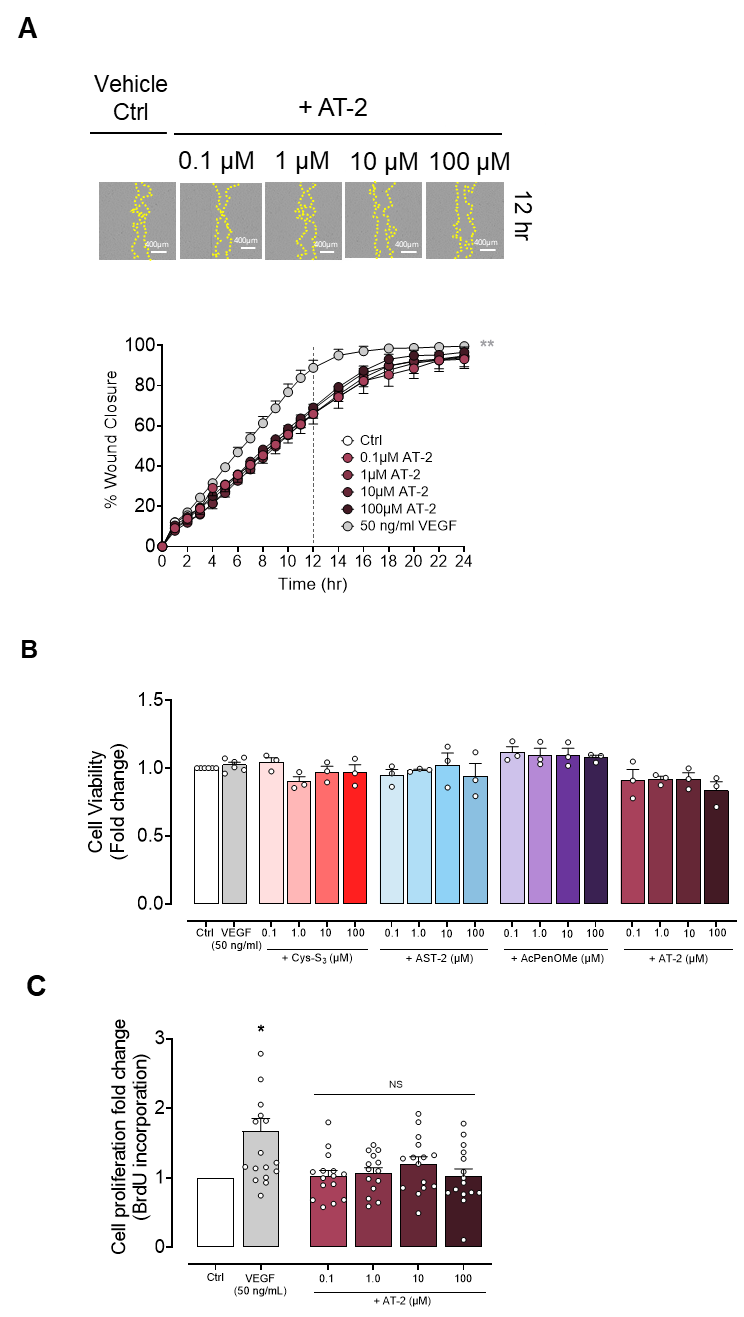


**Supplementary Figure 3**

**Supplementary Figure 4**


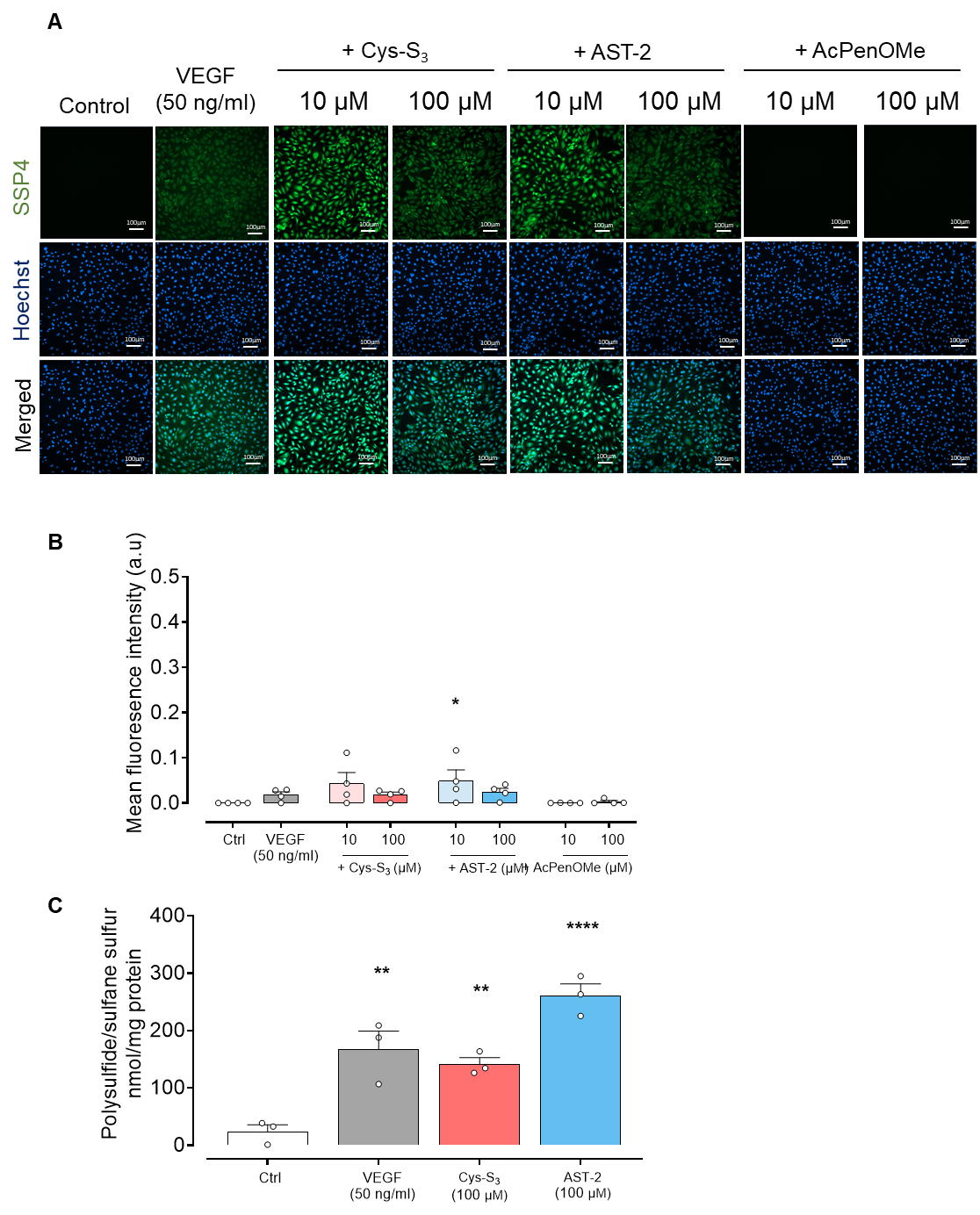


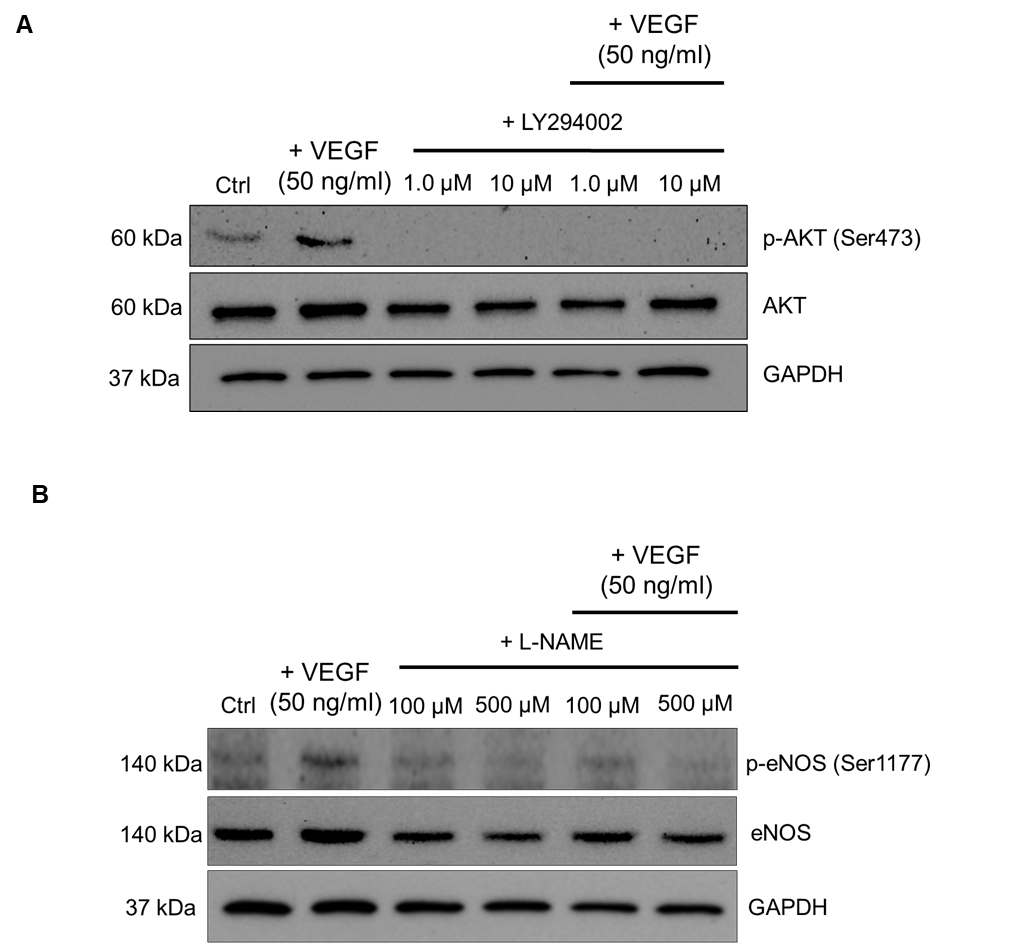


**Supplementary Figure 5**

**Supplementary Figure 6**


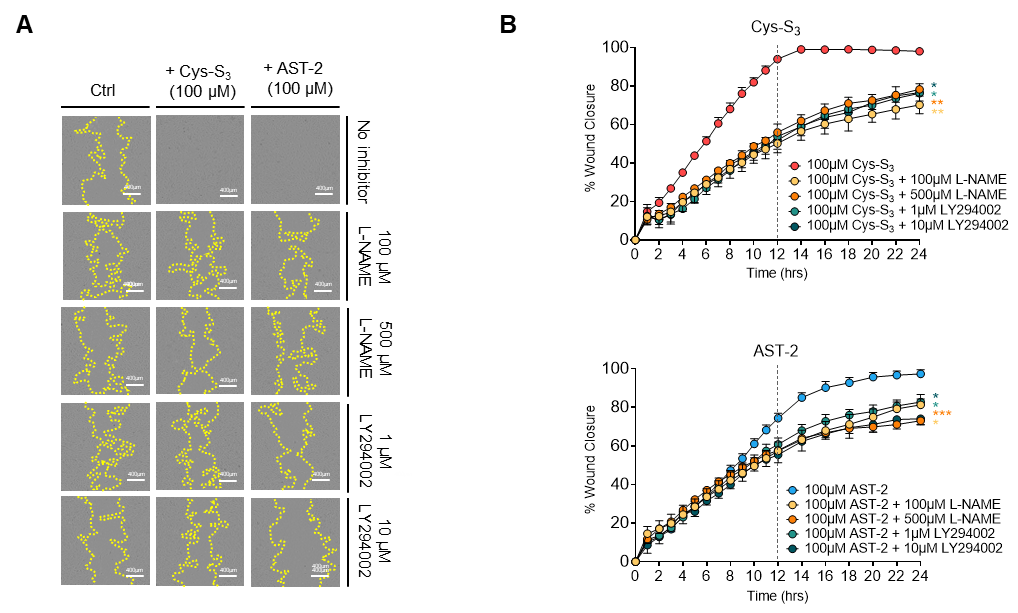


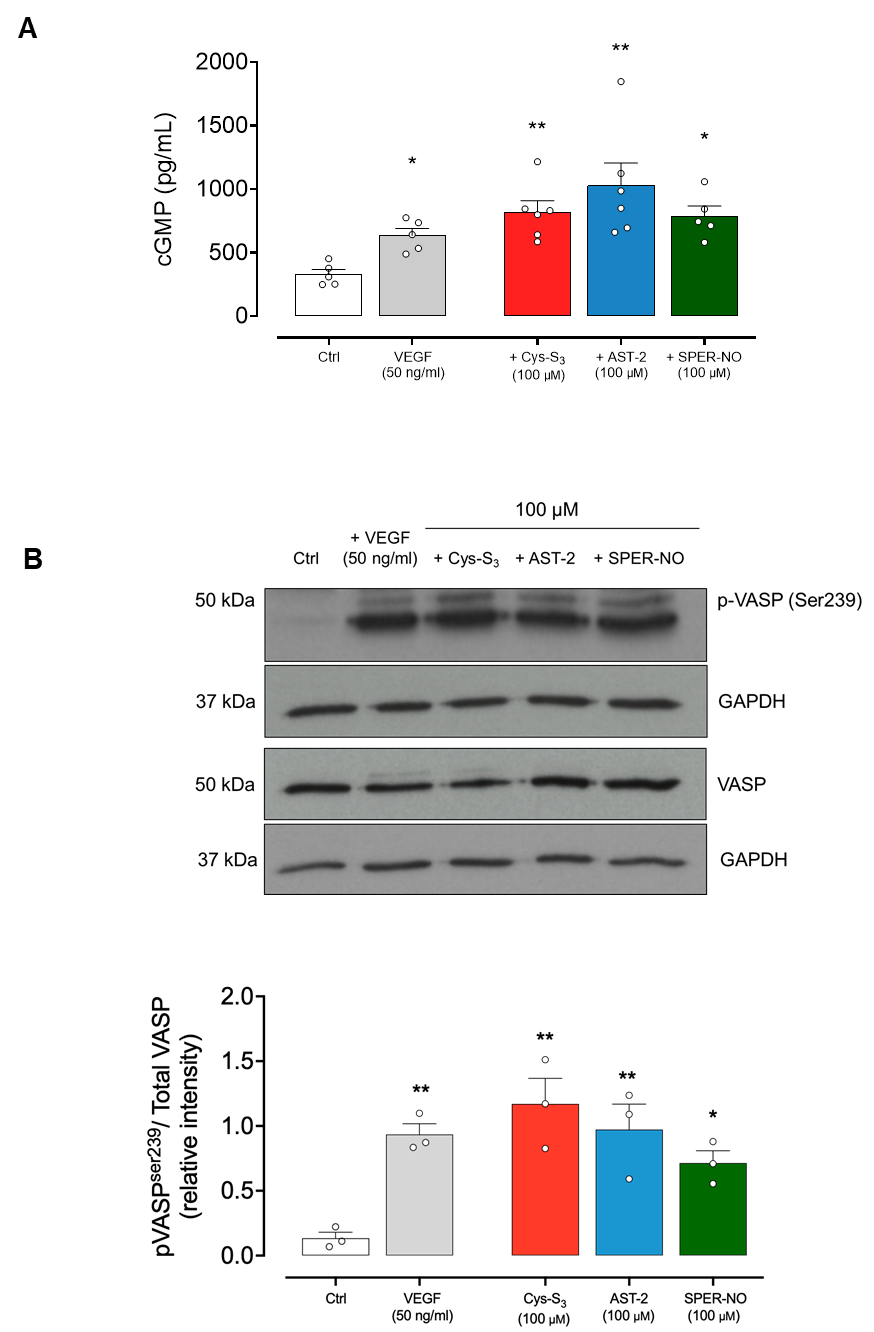


**Supplementary Figure 7**
